# D90 and D_2CC_ Dose in Vaginal CTV as Dosimetric Predictors of Late Vaginal Side Effects in Postoperative Vaginal Modern Brachytherapy (Interventional Radiotherapy) After External Beam Irradiation in Endometrial Cancer

**DOI:** 10.3390/cancers18142350

**Published:** 2026-07-21

**Authors:** Yao Qiang, Faegheh Noorian, Rosa Abellana, Clara Baltrons, Valentina Lancellotta, Luca Tagliaferri, Jaume Ordi, Angeles Rovirosa

**Affiliations:** 1 Fonaments Clínics Department, Faculty of Medicine, Universitat de Barcelona, 08036 Barcelona, Spain; yqiangqi45@alumnes.ub.edu (Y.Q.); faegheh.noorian@gmail.com (F.N.); rabellana@ub.edu (R.A.);; 2 Radiation Oncology Department, Hospital Clínic Universitat de Barcelona, 08036 Barcelona, Spain; baltrons@clinic.cat; 3 Institut d’Investigacions Biomèdiques August Pi i Sunyer (IDIBAPS), C/Casanovas 153, 08036 Barcelona, Spain; 4 Dipartimento di Diagnostica per Immagini, U.O.C. Radioterapia Oncologica, Fondazione Policlinico Universitario A. Gemelli IRCCS, Radioterapia Oncologica ed Ematologia, Università Cattolica del Sacro Cuore, 00168 Rome, Italy; valentina.lancellotta@policlinicogemelli.it (V.L.); luca.tagliaferri@policlinicogemelli.it (L.T.); 5 Barcelona Institute for Global Health, 08036 Barcelona, Spain; 6 Facultat de Medicina i Ciències de la Salut, Universitat de Barcelona, 08036 Barcelona, Spain; 7 Department of Pathology, Hospital Clínic de Barcelona, 08036 Barcelona, Spain; 8 Gynaecological Cancer Unit, Hospital Clinic, Universitat de Barcelona, 08036 Barcelona, Spain

**Keywords:** postoperative endometrial cancer, vaginal-cuff brachytherapy, late vaginal secondary effects, prescription and optimization strategies

## Abstract

**Background/Objectives**: To evaluate clinical and dosimetric predictors of vaginal toxicity in patients treated with vaginal-cuff brachytherapy (VCB), with or without external beam radiotherapy (EBRT), and assess the impact of different prescription and optimization strategies on dose–volume parameters. **Methods**: We retrospectively analyzed 217 patients treated with EBRT combined with VCB (*n* = 120) or VCB alone (*n* = 97). In VCB three prescription and optimization strategies (volume-based, graphical optimization, and 5 mm distance-based prescription) were compared to determine differences in dose–volume parameters. In patients who developed late vaginal side effects, the dosimetric parameters were evaluated using descriptive statistics and Cox regression modeling. Kaplan–Meier analysis was performed to assess time to toxicity. **Results**: In the EBRT+VCB cohort, 34/120 patients (28.3%) developed grade 1–2 vaginal toxicity compared to 35/97 (36%) in the VCB alone cohort. In the EBRT+VCB group, patients with toxicity received a higher D90 per fraction and higher vaginal D_2cc_. In multivariable analysis, vaginal D_2cc_ (HR = 1.60, 95% CI = 1.01–2.54, *p* = 0.045) and CTV-DVH (HR = 0.77, 95% CI = 0.61–0.97, *p* = 0.027) were independently associated with toxicity. No significant dose–toxicity associations were observed in the VCB-alone cohort. Most events occurred within 48 months in both groups. Comparison of prescription strategies showed systematic differences in target and organ-at-risk dose–volume parameters, including vaginal D_2cc_. The 5 mm distance-based prescription consistently resulted in higher D90, D100, EQD2, and vaginal D_2cc_ values. **Conclusions**: In patients receiving EBRT combined with VCB, both vaginal D_2cc_ and target dose distribution were associated with vaginal toxicity. The prescription and optimization strategy significantly influenced dose–volume parameters. Planning methodology and dosimetric aspects should be considered when interpreting dose–toxicity relationships in vaginal brachytherapy.

## Simple Summary

This study investigated dosimetric predictors of vaginal toxicity in patients undergoing vaginal-cuff brachytherapy (VCB), with or without prior external beam radiotherapy (EBRT). In the EBRT+VCB cohort, vaginal D_2cc_ and clinical target volume-dose volume histogram emerged as independent predictors of toxicity, whereas no such correlations were observed in patients treated with VCB alone. Notably, variations in prescription and optimization strategies led to systematic discrepancies in key dose–volume parameters, including vaginal D_2cc_. These findings underscore the profound impact of planning methodology on dosimetric outcomes and the clinical interpretation of dose–toxicity relationships, highlighting the need for standardized approaches in brachytherapy research.

## 1. Introduction

Endometrial cancer (EC) is the most common gynecologic malignancy and the sixth most common cancer among women worldwide [1]. Surgery remains the primary treatment for EC, frequently complemented with radiotherapy. Adjuvant radiotherapy includes external beam radiotherapy (EBRT) and vaginal-cuff brachytherapy (VCB), also called interventional radiotherapy [2]. For women classified as intermediate-risk or high-intermediate-risk, postoperative VCB is the preferred adjuvant approach to ensure optimal local control and prevent vaginal recurrence [3]. Previous studies have reported that postoperative VCB alone can reduce the rate of vaginal recurrence to as low as <2% [4]. Although some guidelines do not consider VCB associated with EBRT, there is no consensus among different centers in Europe, and some hospitals recommend this association based on vaginal, pelvic and distant control [4,5,6,7,8].

In VCB, the most conventional clinical practice is to prescribe the radiation dose at a depth of 0.5 cm (5 mm) from the surface of the applicator. In three-dimensional (3D) image-guided VCB, however, the dose prescription is typically based on coverage of the clinical target volume (CTV). The planning objective is usually to ensure that D90 CTV (the 90% isodose including the CTV) receives the prescribed dose [9]. The use of CTV delineation for VCB, which includes the vaginal wall in the upper vaginal-cuff, has not been well extended in most centers [10].

Since treatment protocols and prescription strategies currently vary across institutions, the dose actually delivered to the CTV may substantially differ [6,11]. In our center, we have observed marked inter-patient variability in vaginal wall thickness. When the dose is prescribed at a fixed depth of 0.5 cm from the applicator surface, variations in wall thickness may lead to significant dose differences, potentially increasing the risk of vaginal toxicity [12].

The organs at risk (OAR) in pelvic radiation therapy include the bladder, rectum, and vagina. The incidence of late rectal and bladder side effects is more closely associated with EBRT, while VCB is more often related to the development of late vaginal side effects (LVSEs), which mainly occur after combined VCB and EBRT treatment. A commonly observed LVSE is Grade-1 (G1) and Grade-2 (G2); VCB- and radiation therapy (RT)-induced in some cases, vaginal stenosis, defined as the abnormal tightening and shortening of the vagina due to the formation of adherences and fibrosis. It is well recognized that advanced RT-induced vaginal stenosis may have a negative impact on patient well-being, especially in relation to sexual dysfunction and dyspareunia and limiting physical examination in post-treatment follow-up. The main factors associated with the development of LVSEs, mainly stenosis, are vaginal surface doses, cylinder diameter, high dose per fraction, active source length, and vaginal dilator (VD) use. However, there is no consensus on the ideal length of time of VD use and dose-fractionation schedules [13,14,15,16,17].

In previous studies by our group, maintaining a total equivalent dose to 2 Gy per fraction (EQD2)(α/β = 3) below 68 Gy to the most exposed 2 cm^3^ of the vagina to the dose, we found a reduction in G2 LVSEs mainly in patients with combined EBRT+VBT [16,18]. A dose constraint of 68 Gy EQD2(α/β = 3) appears to be unnecessary for patients receiving exclusive VCB with standard fractionation schedules of 6 Gy × 3 or 7.5 Gy × 2 as both schedules are equivalent and yield similar rates of LVSEs over time. However, in patients receiving EBRT+VCB a 68 Gy EQD2(α/β = 3) constraint at 2 cm^3^ of the CTV (D_2cc_) and VD use of ≥9 months were independent prognostic factors, having protective effects on LVSEs [19,20,21]. Another previous study compared dosimetry parameters among three different 3D planning strategies for high-dose-rate (HDR) VCB using cylinder applicators postoperatively in EC patients: dose prescription at 5 mm with point optimization (the original prescription), prescription to the CTV with volume point-based optimization and prescription using graphical optimization adapting D90 to the CTV. Volume point-based prescription provided better conformity and reduced the dose to the most exposed 2 cm^3^ of the vagina compared to prescription at 5 mm from the applicator surface. Acceptable results were also obtained using graphical optimization for adapting D90 to CTV [12].

Building on that work, we conducted an expanded analysis involving 217 patients to investigate the relationship between LVSEs and the following dosimetry parameters: CTV volume that receives 100% of the prescribed dose (V100), overall radiation dose delivered to 90% isodose involving the CTV (D90), CTV volume in cc, conformality index (COIN), coverage index (CovI), EQD2 (α/β = 3) ≥ 68 Gy vs. <68 Gy in the most exposed 2 cm^3^ of the vaginal CTV. The objective of the present analysis was also to evaluate how differences in dosimetry parameters among these three prescription strategies are correlated with LVSEs detected when prescription was at 5 mm.

## 2. Materials and Methods

### 2.1. Study Design and Patient Population

This retrospective observational study cohort included 227 EC patients treated with VCB, with or without EBRT, after surgery from 2014 to 2022, with at least three years of follow-up. Patient inclusion required the availability of complete dosimetry data and documented follow-up of toxicity. Ten patients were excluded from the analysis for having a follow-up period of less than 3 years. Thus, we analyzed the dosimetry data of 217 patients.

The present study was approved by the Institutional Ethical Review Board of our hospital, including revision of patient history (HCB/2025/0502).

### 2.2. Treatment

All the patients underwent surgery after the diagnosis of EC, followed by VCB ± EBRT based on the Guidelines of the European Society for Gynecological Oncology- European Society for Treatment in Radiation Oncology, and European Society of Pathology (ESGO-ESTRO-ESP) [8]. Sixty-nine patients received chemotherapy previous to external beam irradiation based on 4–6 cycles of Carboplatin plus Taxol; of the 217 patients, 120 were treated with EBRT followed by one session of VCB with a dose intention of 7 Gy, (5.5–7 Gy). CTV delineation in EBRT followed ASTRO guidelines [12]. EBRT was delivered using 6 or 18 MV photons with fraction doses of 1.8 to 2 Gy per day, 5 days per week. When positive, lymph nodes received a sequential boost dose if 3D techniques were used or a simultaneous boost of 2.2 to 2.4 Gy per fraction when VMAT techniques were implemented (89 patients were treated with 3D-CRT and 31 with VMAT). The remaining 97 patients were treated exclusively with VCB with 2 fractions of 7.5 Gy or 3 fractions of 6 Gy (considered as similar doses). After July 2015, a limit of 68 Gy EQD2(α/β = 3) in the most exposed 2 cm, 3 of CTV (D_2cc_) was administered in 115/217 patients after our previous analysis of vaginal toxicity [12,16,20,21].

### 2.3. High-Dose-Rate VCB

In all the cases in this study HDR VCB was carried out with the vaginal cylinder technique and the Oncentra Brachy planning system v. 4.6 (Elekta^®®^, Nucletron BV, Veenderhaal, The Netherlands) was used for planning. The CTV of the vagina was delineated according to our institutional protocols and described elsewhere. The CTV included the vaginal wall and apex of the upper third of the vagina, typically extending approximately 3 cm and was contoured as a 3 mm ring surrounding the applicator. The contouring of the vagina needed to be corrected by hand in most of the cases to better adapt to the real contour. The active source treatment length was 2.5 cm. Contouring the OAR was carried out slice by slice in the outer contour of the rectum and bladder, from 2 cm above to 2 cm below the CTV [16,18,20,22,23,24,25].

After defining the vaginal CTV and OAR, the same treatment planning was copied to perform three different optimizations for each patient. All patients were originally treated using point-based optimization planning for dose prescription at 5 mm from the applicator. Subsequently, two additional hypothetical treatment plans were generated for each patient using the same CTV, allowing comparison of three different planning and prescription approaches. In the present study, the aim was to evaluate how dosimetry parameters varied among planning techniques and whether these variations may be associated with LVSEs. The three treatment planning possibilities were: T1-arm: dose prescription at 5 mm (the original prescription where the planning details have been reported elsewhere) and in this treatment vaginal toxicity was registered in all the 217 patients; T2-arm: prescription to CTV with volume point-based optimization; and T3-arm, prescription using graphical optimization adapting D90 to the CTV [12,19,20,21,26].

### 2.4. Dosimetry Analysis

Dosimetry parameters were retrospectively collected from the dose–volume histogram (DVH) for all patients to assess their association with LVSEs.

The target volume parameters analyzed included:

• V100 (%): Volume of the CTV receiving 100% of the prescribed dose.
• D100 (Gy): The dose received in 100% isodose of the CTV in a single treatment fraction.
• D90 CTV per fraction (Gy): The radiation dose received by 90% isodose of the CTV in a single treatment fraction.
• Total D90 CTV (Gy): Overall D90 CTV per fraction obtained considering the sum of the number of HDR VCB fractions or EBRT dose plus VBT dose both expressed in EQD2(α/β = 10).
• CTV volume (cc): The total volume of the CTV.
• COIN: The COIN describes how well the reference dose encompasses the CTV and excludes the non-target structures (COIN = C1 × C2, where C1 = V100(CTV)/Volume (CTV) and C2 = V100(CTV)/Vref (Vref being the volume receiving 100% of the prescribed dose irrespective of the target delineated, derived by generating an isodose-structure of volume receiving 100% dose with a posterior automatic exclusion of the cylinder) [27,28].
• CovI: The CovI was defined as the fraction of the CTV that receives the prescribed dose and is an estimate of how much of the CTV received 100% of the dose. CovI = V100(CTV)/Volume (CTV). In ideal conditions, the CI and COIN should be 1 [27,28].
• Vaginal constraint (Gy): Vaginal dose assessment focused on the most exposed 2 cm^3^ of the vaginal CTV, calculated as the equivalent dose in 2 Gy fractions (EQD2) using an α/β ratio of 3. Based on this parameter, patients were stratified into two groups: EQD2 ≥ 68 Gy and EQD2 < 68 Gy.
• The overall EQD2(α/β = 3) in D_2cc_ of vagina was calculated by adding EQD2(α/β = 3) of EBRT to EQD2(α/β = 3) of D_2cc_ of vagina in EBRT+VCB. For exclusive VCB, it was calculated by summing the EQD2(α/β = 3) in D_2cc_ of vagina of all the sessions.
• Vaginal wall thickness was measured at more than 1000 surface points in each patient using a Python (Python version 3.13, 2024) script on the Digital Imaging and Communications in Medicine CTV structure region of interest.
• CTV-DVH (cc): CTV dose volume histogram.

### 2.5. Toxicity Assessment

Treatment-related toxicity was evaluated longitudinally throughout the follow-up period using objective criteria of the Late Effects of Normal Tissues—Subjective, Objective, Management, and Analytic radiation toxicity scale [29]. All recorded toxicities were systematically evaluated and documented by the same specialist and included in the statistical analysis. For G1 these criteria consider the presence of adherences, telangiectasia > 1 cm in extension, stenosis/reduction of vagina less than one third; for G2: bleeding, vaginal stenosis > 1/3 and <2/3; and for G3 a vaginal length < 2/3 of the primary vaginal length.

### 2.6. Statistical Analysis

Patients were stratified into two groups according to treatment modality: those who received EBRT+VCB and those who received VCB alone.

For each group a descriptive and bivariate analysis was performed to evaluate the relationship between toxicity and dosimetry parameters. Descriptive statistics were used to summarize dosimetry variables, and their association with time to toxicity was analyzed and assessed using Cox proportional hazards regression models. Kaplan–Meier analysis was performed to estimate time to vaginal toxicity.

A multivariable Cox model was constructed to assess the independent association between dosimetry parameters and time to toxicity, adjusting for relevant clinical- and treatment-related covariates.

Dosimetry parameters were compared among the three planning/prescription regimens in patients who developed G1 or G2 LVSEs. Comparisons were conducted separately for: patients treated with EBRT+VCB, and patients treated with VCB alone.

A repeated-measure analysis of variance was used to compare differences in mean dosimetry values between regimens, accounting for within-subject correlation of repeated measurements. The assumption of sphericity was evaluated using Mauchly’s test. When the assumption of sphericity was violated, the Samuel Greenhouse–Seymour Geisser correction was applied. Post hoc pairwise comparisons between regimens were conducted using estimated marginal means, with Bonferroni adjustment for multiple comparisons. Adjusted *p*-values were reported for all pairwise contrasts. A *p*-value < 0.05 was considered statistically significant. Analyses were performed using R statistical software (R Core Team V. 4.5.3)), and repeated-measure analyses were conducted using the rstatix package [30,31].

## 3. Results

A total of 217 patients were included in the study. Among these, 120 patients received EBRT+VCB and 97 patients were treated with exclusive VCB. The results are presented separately according to each treatment group.

### 3.1. Results in the EBRT+VCB Group

In the EBRT+VCB group (*n* = 120), 34 (28.3%) patients developed G1–2 LVSEs (Table 1). G1 appeared in 22/34 (65%), it being 26.4% of this group; G2 complications were found in 12/34 (35%) patients with LVSE, it being the 14% of the entire group. Descriptive analysis showed that patients with LVSEs had a significantly higher D90 CTV per fraction (7.91 ± 0.39 vs. 7.71 ± 0.65, *p* = 0.040) and a higher vaginal D_2cc_ compared with patients without toxicity (10.1 ± 0.60 vs. 9.84 ± 0.76, *p* = 0.045). In the present analysis there was little compliance with the use of VDs for more than 9 months; and there was a tendency of applying a D_2cc_ dose > 68 Gy EQD2(α/b = 3) (*p* = 0.055).

**Figure 1:**
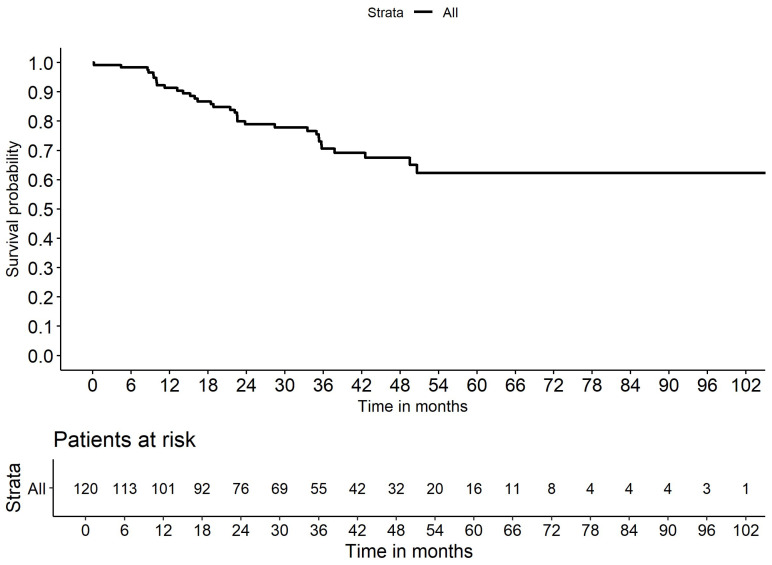
Probability of time free of late vaginal side effects in the EBRT+VCB cohort.

Kaplan–Meier analysis showed that most events occurred within the first 48 months of follow-up, followed by a plateau thereafter (Figure 1).

No significant associations were observed in the univariate Cox analyses (Table 2). However, in the multivariable model, both CTV-DVH and vaginal D_2cc_ emerged as independent predictors of toxicity: a higher CTV-DVH was associated with a reduced risk (hazard ratio [HR] = 0.77, 95% confidence interval [CI]: 0.61–0.97; *p* = 0.027), whereas a higher vaginal D_2cc_ was associated with an increased risk (HR = 1.60, 95% CI: 1.01–2.54; *p* = 0.045). This likely reflects modest effect sizes and limited statistical power in univariate analyses, with improved precision after joint modeling. Bootstrap resampling confirmed the stability of these associations, with percentile confidence intervals that did not cross the null value for either variable.

### 3.2. Results in the Exclusive VCB Group

**Figure 2:**
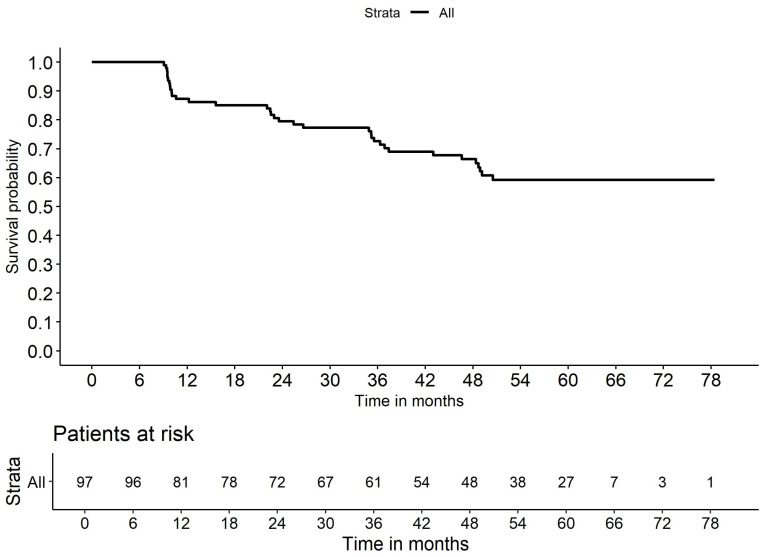
Probability of time free of late vaginal complications in the VCB alone group.

Kaplan–Meier analysis showed a similar temporal distribution of events, with most toxicities occurring within the first 48 months (Figure 2).

In the exclusive VCB group (*n* = 97), 35 (36%) patients developed grade 1–2 LVSEs. G1 side effects appeared in 24/35 (69%) patients and 25.77% in the overall group, G2 side effects appeared in 11/35 (31%) and 11.34% in the overall group. Descriptive analysis did not reveal any statistically significant differences between dosimetric parameters and the occurrence of LVSEs. Similarly, univariate Cox regression analysis did not identify any variables significantly associated with time to toxicity (Table 3 and Table 4).

### 3.3. The Three Prescription Approaches Compared Among Patients Who Developed G1 1–2 Vaginal Secondary Effects

Among EBRT+VCB patients with LVSEs (*n* = 34), all comparisons demonstrated significant differences among the three strategies in D90 CTV per fraction, total D90 CTV, D100, CovI, COIN, vaginal D_2cc_, and total EQD2(α/β = 3) whereas the CTV volume did not differ (Table 5). This has been previously reported by our group [12].

Among exclusive VCB patients with toxicity (*n* = 35), the three prescription strategies also showed significant differences in D90 CTV per fraction, total D90 CTV, D100, CI, COIN, vaginal D_2cc_, and total EQD2(α/β = 3) (Table 6).

## 4. Discussion

In this study, we evaluated dosimetric and clinical predictors of vaginal toxicity in patients treated with EBRT+VCB and in those treated with VCB alone, and we further explored how different brachytherapy prescription strategies can influence dose–volume parameters in patients who developed LVSEs.

### 4.1. Predictors of Vaginal Toxicity in the EBRT+VCB Group

In the EBRT+VCB cohort, patients who developed grade 1–2 vaginal toxicity received a significantly higher D90 CTV per fraction and higher vaginal D_2cc_ compared with those without toxicity. While D90 CTV primarily reflects target coverage, vaginal D_2cc_ represents focal OAR exposure and has been widely used as a surrogate for LVSEs. The association between increased vaginal D_2cc_ and toxicity is therefore consistent with established dose–response relationships for late vaginal morbidity. The volume of the CTV can vary among patients and consequently, the dose received by the CTV can differ. In univariate analysis, the D90 CTV per fraction was also higher among patients with vaginal toxicity (*p* = 0.40). Moreover, given the steep dose gradients characteristic of vaginal brachytherapy, escalation of target dose may be accompanied by increased dose deposition in adjacent vaginal tissues. Thus, the D90 CTV may also reflect the overall local dose to healthy neighboring structures in addition to an isolated CTV effect. In addition, smaller CTV volumes may receive relatively higher dose levels, which in our analysis were associated with increased toxicity [12]. The D90 CTV can be adapted to CTV needs using graphical optimization or prescription to volume depending on the case. A previous study by our group suggested that a D90 CTV of approximately 7.27 Gy may represent an appropriate target dose when graphical optimization is used [11,12].

Vaginal dilator use was not significantly associated with vaginal toxicity in the EBRT+VCB cohort in the univariate analysis. Although the proportion of dilator use was lower among patients who developed toxicity, the overall number of patients using dilators was limited, which, in the present study, may have reduced the ability to detect the association that has been reported in other studies [20,21]. Consequently, considering the literature, the use of VDs should be encouraged whenever possible, ideally starting within the first 2 weeks after brachytherapy and continuing for at least 4 years. The same evolution over time in LVSEs was observed in the VCB alone group, and thus, the same VD policy should be applied to this group.

In the present analysis, vaginal D_2cc_ was not statistically significant in univariate Cox analysis but a trend was observed. In the multivariate model, which incorporated representative parameters of target coverage and OAR dose, both CTV-DVH and vaginal D_2cc_ emerged as independent factors associated with LVSEs. A higher CTV-DVH was associated with a reduced risk of LVSEs (HR = 0.77, 95% CI 0.61–0.97, *p* = 0.027), whereas a higher vaginal D_2cc_ was associated with an increased risk (HR = 1.60, 95% CI 1.01–2.54, *p* = 0.045). These findings suggest that target coverage and focal organ-at-risk exposure represent complementary dimensions of treatment planning that may jointly influence the risk of vaginal toxicity [11,12,20,21]. Consequently, adequate D90 CTV prescription targeting using geometric optimization together with a D_2cc_ constraint of <68 Gy EQD2(α/β = 3) may represent a reasonable planning approach for these patients to reduce LVSEs.

### 4.2. Predictors of Vaginal Toxicity in the VCB Alone Group

In contrast, in the VCB alone cohort, no statistically significant associations were observed between dosimetric parameters and either toxicity occurrence or timing. Although the temporal pattern of events was similar to that of the EBRT+VCB cohort, no dominant dosimetric predictor was identified. This may be related to the fact that this group of patients received a much lower dose than the EBRT+BT group. As shown in Table 1 the dose per fraction is between 7.82 and 7.91 Gy for combined EBRT+VCB but in VCB alone the dose is 7 Gy x2 showing the diference in the dose received by the two tratment groups. Although the overall EQD2(α/β = 3) was greater in the EBRT+BT group (*p* = 0.00), the number of G1-G2 LVSEs was similar to the VCB alone group and this may be related to the low compliance in the use of VDs in both groups. Interindividual variations in radiotherapy dose may also have an effect on the incidence of side effects that can explain the behavior between the two groups. Another possibility is that these findings on LVSEs may appear only after the increase in a concrete dose in D_2cc_, D90 CTV or in vaginal mucosa.

### 4.3. Impact of Prescription and Optimization Strategies

A key finding of this study is that different brachytherapy prescription and optimization strategies resulted in systematic differences in target dose metrics and OAR parameters, in a setting of identical CTV volumes in each group. In both treatment arms (EBRT+VCB and exclusive VCB), the 5 mm distance-based prescription consistently yielded a higher D90 CTV, D100, EQD2, and vaginal D_2cc_ compared with volume-based and graphical optimization approaches.

From a methodological perspective, this observation is particularly relevant. In the EBRT+VCB cohort, vaginal D_2cc_ was an independent predictor of side effects in contrast to exclusive VCB patients [16,20,22,23,25]. This last aspect is related to the fact that in the exclusive VCB group the dose received was lower and did not surpass the D_2cc_ constraint. In these patients, it is likely that other dosimetric parameter values may have a greater influence on the appearance of LVSEs in addition to the lack of adherence to the use of VDs.

Moreover, our dosimetric comparison demonstrates that vaginal D_2cc_ itself varies according to the prescription and optimization strategy. Consequently, the numerical value of a clinically relevant side effect predictor may differ depending on planning methodology, even when patient anatomy and applicator geometry remain unchanged. This has implications for dose–response modeling, risk stratification, and inter-institutional comparisons in gynecologic brachytherapy. Vaginal wall thickness varies among patients and although the mean is 3 mm, there is a variation among patients when manual correction of delimitation is performed. This last comment is in line with recommending D90 CTV graphical optimization as a first procedure in these patients followed by D90 CTV prescription, and finally, the 5 mm prescription from the applicator [12].

This study has some limitations: The retrospective nature of the study limits causal inference and introduces the possibility of selection bias. Although the number of toxicity events allowed multivariable analysis, the sample size—particularly in the VCB alone cohort—may have limited the detection of smaller effect sizes. The analysis was restricted to grade 1–2 vaginal toxicity, and more severe late complications may demonstrate different dose–response characteristics. In addition, the dosimetric comparison was confined to three of the most common applied prescription and optimization strategies and other planning approaches were not evaluated, which may limit broader generalizability.

## 5. Conclusions

In the EBRT+VCB cohort, vaginal D_2cc_ and CTV-DVH were independently associated with vaginal toxicity, highlighting the clinical relevance of both OAR dose and target coverage. No significant dose–toxicity associations were identified in the VCB alone group. This is the first time this has been reported in the literature.

The prescription and optimization strategy systematically influenced dose–volume parameters, including vaginal D_2cc_, despite unchanged CTV volumes. These findings underscore the importance of planning methodology in interpreting dose–response relationships and comparing outcomes across institutions.

The present study opens the hypothesis that VCB dose prescription should be adapted to the CTV using geometrical or volume prescription. Thus, prospective studies are strongly warranted.

Finally, the use of VDs should be strongly recommended.

## Figures and Tables

**Table 1:** Descriptive and bivariate analysis of late vaginal side effects according to dosimetric parameters in EBRT+VCB.

	ALL	NO	G.I–II	*p*-Value	*n*
	*n* = 120	*n* = 86	*n* = 34		
Age	64.9 (10.6)	65.6 (10.2)	63.0 (11.3)	0.242	120
D90 CTV per fraction (Gy)	7.77 (0.59)	7.71 (0.65)	7.91 (0.39)	0.040	120
Total D90 CTV (Gy)(EBRT+VCB)	7.84 (0.75)	7.82 (0.85)	7.91 (0.39)	0.404	120
D100 (Gy)	6.57 (0.72)	6.54 (0.78)	6.65 (0.56)	0.391	120
CovI	0.99 (0.05)	0.98 (0.06)	1.00 (0.01)	0.079	120
COIN	0.37 (0.07)	0.38 (0.07)	0.37 (0.06)	0.433	120
Vagina D_2cc_ (Gy)	9.92 (0.72)	9.84 (0.76)	10.1 (0.60)	0.045	120
Total EQD2(α/b = 3) (Gy)	70.1 (5.31)	69.9 (5.91)	70.8 (3.34)	0.265	120
D_2cc_ EQD2(α/b = 3) ≤ 68 Gy	50 (41.7%)	41 (47.7%)	9 (26.5%)	0.055	120
D_2cc_ EQD2(α/b = 3) > 68 Gy	70 (58.3%)	45 (52.3%)	25 (73.5%)		
CTV-DVH (cc)	8.07 (1.50)	8.22 (1.55)	7.70 (1.30)	0.067	120
Vaginal min. thickness (mm) Mean (SD)	0.36 (0.48)	0.38 (0.49)	0.32 (0.45)	0.587	118
Vaginal max. thickness					
(mm) Mean (SD)	6.04 (1.62)	5.95 (1.77)	6.29 (1.14)	0.225	118
Vaginal mean thickness					
(mm) Mean (SD)	3.09 (0.48)	3.10 (0.50)	3.05 (0.42)	0.576	118
Vaginal median thickness					
(mm) Mean (SD)	3.17 (0.45)	3.18 (0.45)	3.13 (0.45)	0.565	118
Vaginal dilator use > 9 months:				0.432	111
No	103 (92.8%)	71 (91.0%)	32 (97.0%)		
Yes	8 (7.21%)	7 (8.97%)	1 (3.03%)		
Cylinder diameter (cm):				0.809	119
2.5	4 (3.36%)	3 (3.53%)	1 (2.94%)		
3	15 (12.6%)	12 (14.1%)	3 (8.82%)		
3.5	100 (84.0%)	70 (82.4%)	30 (88.2%)		

D90 (Gy): Dose received by 90% of the clinical target volume (CTV). Total D90 (Gy): Cumulative D90 dose delivered to the CTV, accounting for all treatment components, expressed in Gy (EQD2). D100 dose (Gy): Minimum dose received by 100% of the CTV. CovI (Coverage Index): Quantitative measure of the conformity between the prescription isodose volume and the target volume. COIN (Conformity Index with Organs at Risk): Index assessing target dose conformity while incorporating dose exposure to organs at risk. Vaginal D_2cc_ (Gy): Dose received by the most exposed 2 cm^3^ of vaginal tissue. Total EQD2(α/β = 3) (Gy): Total biologically equivalent dose in 2-Gy fractions calculated using an α/β ratio of 3 Gy. CTV (Clinical Target Volume): Volume encompassing the tumor bed and regions at risk of microscopic disease. CTV-DVH: CTV dose–volume histogram; SD: standard deviation.

**Table 2:** Association between dosimetric parameters and time to late vaginal side effects in EBRT+VCB (Univariate analysis).

	No Event	Event	HR [95% CI]	*p*-Value	*n*
	*n* = 86	*n* = 34			
D90 CTV per fraction (Gy)	7.71 (0.65)	7.91 (0.39)	1.82 [0.96; 3.46]	0.068	120
D100 (Gy)	6.54 (0.78)	6.65 (0.56)	1.26 [0.76; 2.10]	0.376	120
CovI	0.98 (0.06)	1.00 (0.01)	-	0.233	120
COIN	0.38 (0.07)	0.37 (0.06)	0.09 [0.00; 17.7]	0.368	120
CTV-DVH (cc)	8.22 (1.55)	7.70 (1.30)	0.82 [0.65; 1.02]	0.080	120
Total EQD2(α/β = 3) (Gy)	69.9 (5.91)	70.8 (3.34)	1.01 [0.96; 1.07]	0.600	120
Total EQD2(α/β = 3) (Gy)					120
D_2cc_ EQD2(α/b = 3) ≤ 68 Gy	41 (47.7%)	9 (26.5%)	Ref.	Ref.	
D_2cc_ EQD2(α/b = 3) > 68 Gy	45 (52.3%)	25 (73.5%)	1.97 [0.92; 4.22]	0.082	
Vaginal thickness (mm):					
Vaginal min. thickness (mm) Mean (SD)	0.38 (0.49)	0.32 (0.45)	0.85 [0.37; 1.98]	0.707	118
Vaginal max. thickness (mm) Mean (SD)	5.95 (1.77)	6.29 (1.14)	1.05 [0.88; 1.25]	0.585	118
Vaginal mean thickness					
(mm) Mean (SD)	3.10 (0.50)	3.05 (0.42)	0.75 [0.36; 1.58]	0.451	118
Vaginal median thickness					
(mm) Mean (SD)	3.18 (0.45)	3.13 (0.45)	0.70 [0.32; 1.55]	0.384	118
Vaginal Dilator use > 9 months:					111
No	71 (91.0%)	32 (97.0%)	Ref.	Ref.	
Yes	7 (8.97%)	1 (3.03%)	0.37 [0.05; 2.69]	0.325	
Applicator diameter (cm):					119
2.5	3 (3.53%)	1 (2.94%)	Ref.	Ref.	
3	12 (14.1%)	3 (8.82%)	0.95 [0.10; 9.18]	0.965	
3.5	70 (82.4%)	30 (88.2%)	1.49 [0.20; 11.0]	0.694	

D90 (Gy): Dose received by 90% of the clinical target volume (CTV). Total D90 (Gy): Cumulative D90 dose delivered to the CTV, accounting for all treatment components, expressed in Gy (EQD2). D100 dose (Gy): Minimum dose received by 100% of the CTV. CovI (Coverage Index): Quantitative measure of the conformity between the prescription isodose volume and the target volume. COIN (Conformity Index with Organs at Risk): Index assessing target dose conformity while incorporating dose exposure to organs at risk. Vaginal D_2cc_ (Gy): Dose received by the most exposed 2 cm^3^ of vaginal tissue. Total EQD2(α/β = 3) (Gy): Total biologically equivalent dose in 2-Gy fractions calculated using an α/β ratio of 3 Gy. CTV (Clinical Target Volume): Volume encompassing the tumor bed and regions at risk of microscopic disease. CTV-DVH: CTV dose–volume histogram; SD: standard deviation.

**Table 3:** Descriptive and bivariate analysis of toxicity according to dosimetric parameters in exclusive VCB (Mean and SD).

	ALL	NO	G.I–II	*p*-Value	*n*
	*n* = 97	*n* = 62	*n* = 35		
Age	65.8 (9.19)	65.5 (9.57)	66.3 (8.56)	0.649	97
D90 CTV per fraction (Gy)	8.07 (1.09)	8.14 (1.07)	7.95 (1.14)	0.420	97
Total D90 CTV (Gy)	18.3 (1.98)	18.2 (1.71)	18.5 (2.41)	0.485	97
D100 dose (Gy)	6.83 (1.15)	6.83 (1.18)	6.83 (1.12)	0.987	97
CovI	0.99 (0.02)	0.99 (0.02)	0.99 (0.03)	0.907	97
COIN	0.37 (0.06)	0.37 (0.07)	0.38 (0.06)	0.649	97
Vagina D_2cc_	10.2 (1.15)	10.3 (1.15)	9.99 (1.15)	0.175	97
Total EQD2(α/β = 3) (Gy)	61.1 (6.32)	61.5 (6.03)	60.4 (6.85)	0.422	97
EQD2(α/β = 3) (Gy):				0.150	97
D_2cc_ EQD2(α/b = 3) ≤ 68 Gy	88 (90.7%)	54 (87.1%)	34 (97.1%)		
D_2cc_ EQD2(α/b = 3) > 68 Gy	9 (9.28%)	8 (12.9%)	1 (2.86%)		
CTV-DVH(cc)	8.13 (1.56)	8.18 (1.60)	8.04 (1.51)	0.678	97
Vaginal min. thickness (mm) Mean (SD)	0.42 (0.48)	0.45 (0.48)	0.35 (0.50)	0.336	97
Vaginal max. thickness					
(mm)	5.93 (1.72)	6.12 (1.86)	5.58 (1.39)	0.108	97
Vaginal mean thickness					
(mm)	3.05 (0.45)	3.07 (0.44)	3.00 (0.49)	0.504	97
Vaginal median thickness					
(mm)	3.12 (0.43)	3.12 (0.41)	3.11 (0.47)	0.944	97
Vaginal Dilator use > 9 months:				0.790	85
No	61 (71.8%)	37 (69.8%)	24 (75.0%)		
Yes	24 (28.2%)	16 (30.2%)	8 (25.0%)		
Cylinder diameter (cm):				0.520	97
2.5	3 (3.09%)	2 (3.23%)	1 (2.86%)		
3	13 (13.4%)	7 (11.3%)	6 (17.1%)		
3.5	80 (82.5%)	53 (85.5%)	27 (77.1%)		
copostat3	1 (1.03%)	0 (0.00%)	1 (2.86%)		

D90 (Gy): Dose received by 90% of the clinical target volume (CTV). Total D90 (Gy): Cumulative D90 dose delivered to the CTV, accounting for all treatment components, expressed in Gy (EQD2). D100 dose (Gy): Minimum dose received by 100% of the CTV. CovI (Coverage Index): Quantitative measure of the conformity between the prescription isodose volume and the target volume. COIN (Conformity Index with Organs at Risk): Index assessing target dose conformity while incorporating dose exposure to organs at risk. Vaginal D_2cc_ (Gy): Dose received by the most exposed 2 cm^3^ of vaginal tissue. Total EQD2(α/β = 3) (Gy): Total biologically equivalent dose in 2-Gy fractions calculated using an α/β ratio of 3 Gy. CTV (Clinical Target Volume): Volume encompassing the tumor bed and regions at risk of microscopic disease. CTV-DVH (cc): CTV dose volume histogram; SD: standard deviation.

**Table 4:** Association between dosimetric parameters and time to late vaginal side effects in exclusive VCB.

	No Event	Event	HR [95% CI]	*p*-Value	*n*
	*n* = 62	*n* = 35			
D90 CTV per fraction (Gy)	8.14 (1.07)	7.95 (1.14)	0.95 [0.71; 1.28]	0.745	97
Total D90 CTV	18.2 (1.71)	18.5 (2.41)	1.06 [0.89; 1.25]	0.520	97
D100 dose (Gy)	6.83 (1.18)	6.83 (1.12)	1.06 [0.80; 1.41]	0.695	97
CovI	0.99 (0.02)	0.99 (0.03)	-	0.584	97
COIN	0.37 (0.07)	0.38 (0.06)	2.70 [0.02; 404]	0.697	97
CTV-DVH (cc)	8.18 (1.60)	8.04 (1.51)	0.96 [0.78; 1.18]	0.698	97
Vagina D_2cc_	10.3 (1.15)	9.99 (1.15)	0.89 [0.68; 1.16]	0.378	97
Total EQD2(α/β = 3) (Gy)	61.5 (6.03)	60.4 (6.85)	0.98 [0.94; 1.03]	0.493	97
Total EQD2(α/β = 3) (Gy)					97
D_2cc_ EQD2(α/b = 3) ≤ 68 Gy	54 (87.1%)	34 (97.1%)	Ref.	Ref.	
D_2cc_ EQD2(α/b = 3) > 68 Gy	8 (12.9%)	1 (2.86%)	0.21 [0.03; 1.51]	0.120	
Vaginal thickness (mm):					
Vaginal min. thickness (mm) Mean (SD)	0.45 (0.48)	0.35 (0.50)	0.70 [0.29; 1.68]	0.425	97
Vaginal max. thickness (mm) Mean (SD)	6.12 (1.86)	5.58 (1.39)	0.84 [0.68; 1.05]	0.119	97
Vaginal mean thickness					
(mm) Mean (SD)	3.07 (0.44)	3.00 (0.49)	0.68 [0.31; 1.48]	0.334	97
Vaginal median thickness					
(mm) Mean (SD)	3.12 (0.41)	3.11 (0.47)	0.86 [0.40; 1.88]	0.714	97
Vaginal Dilator use > 9 months:					85
No	37 (69.8%)	24 (75.0%)	Ref.	Ref.	
Yes	16 (30.2%)	8 (25.0%)	0.78 [0.35; 1.73]	0.541	
Applicator diameter (cm):					97
2.5	2 (3.23%)	1 (2.86%)	Ref.	Ref.	
3	7 (11.3%)	6 (17.1%)	1.06 [0.13; 8.77]	0.960	
3.5	53 (85.5%)	27 (77.1%)	0.85 [0.12; 6.28]	0.876	
copostat3	0 (0.00%)	1 (2.86%)	7.77 [0.46; 131]	0.155	

D90 (Gy): Dose received by 90% of the clinical target volume (CTV). Total D90 (Gy): Cumulative D90 dose delivered to the CTV, accounting for all treatment components, expressed in Gy (EQD2). D100 dose (Gy): Minimum dose received by 100% of the CTV. CovI (Coverage Index): Quantitative measure of the conformity between the prescription isodose volume and the target volume. COIN (Conformity Index with Organs at Risk): Index assessing target dose conformity while incorporating dose exposure to organs at risk. Vaginal D_2cc_ (Gy): Dose received by the most exposed 2 cm^3^ of vaginal tissue. Total EQD2(α/β = 3) (Gy): Total biologically equivalent dose in 2-Gy fractions calculated using an α/β ratio of 3 Gy. CTV (Clinical Target Volume): Volume encompassing the tumor bed and regions at risk of microscopic disease. CTV-DVH (cc): CTV dose volume histogram; SD: standard deviation.

**Table 5:** EBRT+VCB: Comparison of dosimetric parameters in the 3 prescription regimes among patients with GI-II.

				Mean Differences [95% CI]		
	Volume	Graphical	5 mm	Volume vs. Graphical	Volume vs. 5 mm	Graphical vs. 5 mm
	*n* = 34	*n* = 34	*n* = 34			
D90 CTV per fraction (Gy) *	7.79 (0.51)	7.37 (0.46)	7.91 (0.39)	0.42 * [0.14, 0.69]	−0.12 [−0.28,0.03]	−0.54 * [−0.80, −0.28]
D100 dosis Gy *	6.35 (0.68)	6.29 (0.31)	6.65 (0.56)	0.06 [−0.26, 0.38]	−0.30 * [−0.50, −0.11]	−0.37 * [−0.64, −0.10]
CovI *	0.91 (0.03)	0.98 (0.07)	1.01 (0.02)	−0.07 * [−0.11, −0.04]	−0.10 * [−0.12, −0.09]	−0.03 [−0.06, 0.00]
COIN *	0.51 (0.12)	0.52 (0.15)	0.38 (0.06)	0.00 [−0.07, 0.06]	0.14 * [0.08, 0.19]	0.14 * [ 0.08, 0.20]
Vagina D_2cc_ *	9.75 (0.88)	9.47 (1.17)	10.1 (0.60)	0.28 [−0.23, 0.79]	−0.36 * [−0.69, −0.03]	−0.64 * [ −1.04, −0.24]
Total EQD2(α/β = 3) *	69.3 (4.06)	68.1 (5.50)	70.8 (3.34)	1.12 [−1.05, 3.30]	−1.56 * [−2.90, −0.22]	−2.69 * [−4.46, −0.91]

* *p*-value < 0.05, D90: Dose received by 90% of the clinical target volume (CTV). Total D90 (Gy): Cumulative D90 dose delivered to the CTV, accounting for all treatment components, expressed in Gy (EQD2). D100 dose (Gy): Minimum dose received by 100% of the CTV. CovI (Coverage Index): Quantitative measure of the conformity between the prescription isodose volume and the target volume. COIN (Conformal Index with Organs at Risk): Index assessing target dose conformity while incorporating dose exposure to organs at risk. Vaginal D_2cc_ (Gy): Dose received by the most exposed 2 cm^3^ of vaginal tissue. Total EQD2(α/β = 3) (Gy): Total biologically equivalent dose in 2-Gy fractions calculated using an α/β ratio of 3 Gy. CTV (Clinical Target Volume): Volume encompassing the tumor bed and regions at risk of microscopic disease; CI: confidence interval.

**Table 6:** Exclusive VCB: Comparison of dosimetric parameters in the 3 prescription regimes that developed late vaginal side effects.

	Volume	Graphical	5 mm	Volume vs. Graphical	Volume vs. 5 mm	Graphical vs. 5 mm
	*n* = 35	*n* = 35	*n* = 35			
D90 CTV per fraction *	7.94 (1.14)	7.21 (0.84)	7.95 (1.14)	0.72 * [0.36, 1.09]	−0.01 * [−0.02,0.00]	−0.73 * [−1.10, −0.37]
Total D90 CTV of VCB *	18.5 (2.41)	17.0 (2.88)	18.5 (2.41)			
D100 dose (Gy) *	6.68 (1.10)	6.21 (0.65)	6.83 (1.12)	0.47 * [0.16, 0.78]	−0.16 * [−0.27, −0.04]	−0.62 * [−0.95, −0.29]
CovI *	0.92 (0.03)	0.96 (0.07)	1.00 (0.03)	−0.04 * [−0.07, −0.01]	−0.09 * [−0.10, −0.07]	−0.05 * [−0.08, 0.01]
COIN *	0.56 (0.11)	0.59 (0.17)	0.38 (0.06)	−0.03 [−0.11, 0.04]	0.18 * [0.12, 0.23]	0.21 * [ 0.14, 0.29]
Vagina 2cc *	9.62 (1.53)	9.13 (1.36)	9.99 (1.15)	0.50 [−0.20, 1.19]	−0.36 * [−0.67, −0.05]	−0.86 * [ −1.38, −0.34]
Total EQD2(α/β = 3) *	56.6 (10.0)	52.7 (14.8)	60.4 (6.85)	3.87 [−3.73, 11.47]	−3.82 * [−7.06, −0.58]	−7.69 * [−13.51, −1.86]

* *p*-value < 0.05, D90: Dose received by 90% of the clinical target volume (CTV). Total D90 (Gy): Cumulative D90 dose delivered to the CTV, accounting for all treatment components, expressed in Gy (EQD2). D100 dose (Gy): Minimum dose received by 100% of the CTV. CovI (Coverage Index): Quantitative measure of the conformity between the prescription isodose volume and the target volume. COIN (Conformal Index with Organs at Risk): Index assessing target dose conformity while incorporating dose exposure to organs at risk. Vaginal D_2cc_ (Gy): Dose received by the most exposed 2 cm^3^ of vaginal tissue. Total EQD2(α/β = 3) (Gy): Total biologically equivalent dose in 2-Gy fractions calculated using an α/β ratio of 3 Gy. CTV (Clinical Target Volume): Volume encompassing the tumor bed and regions at risk of microscopic disease; CI: confidence interval.

## Data Availability

The data presented in this study are available on reasonable request from the corresponding author.
